# From Clinical Diagnosis to the Discovery of Multigene Rare Sequence Variants in *Pseudoxanthoma elasticum*: A Case Report

**DOI:** 10.3389/fmed.2021.726856

**Published:** 2021-08-26

**Authors:** Francesco Demetrio Lofaro, Dario Pasquale Mucciolo, Vittoria Murro, Laura Pavese, Daniela Quaglino, Federica Boraldi

**Affiliations:** ^1^Department of Life Science, University of Modena and Reggio Emilia, Modena, Italy; ^2^Department of Neuroscience, Psychology, Drug Research and Child Health, University of Florence, Eye Clinic, Florence, Italy

**Keywords:** ABCC6, calcification, PXE, rare disease, skin

## Abstract

*Pseudoxanthoma elasticum* (PXE) is a rare autosomal recessive disease clinically characterised by early cutaneous alterations, and by late clinically relevant ocular, and cardiovascular manifestations. *ABCC6* genetic tests are used to confirm clinical PXE diagnosis, but this strategy may be rather challenging when only one *ABCC6* pathogenic variant is found. A next-generation sequencing approach focusing on 362 genes related to the calcification process and/or to inherited retinal diseases was performed on a patient with clinical PXE diagnosis (skin papules and laxity, angioid streaks, and atrophy) who was carrier of only one *ABCC6* rare sequence variant. Beside *ABCC6*, several rare sequence variants were detected which can contribute either to the occurrence of calcification (*GGCX* and *SERPINF1* genes) and/or to ophthalmological manifestations (*ABCA4, AGBL5, CLUAP1*, and *KCNV2* genes). This wide-spectrum analysis approach facilitates the identification of rare variants possibly involved in PXE, thus avoiding invasive skin biopsy as well as expensive and time-consuming diagnostic odyssey and allows to broaden and to deepen the knowledge on this complex rare disease and to improve patients' counselling, also with a future perspective of personalised medicine.

## Introduction

*Pseudoxanthoma elasticum* (PXE; OMIM#264800) is an inherited disorder characterised by calcified elastic fibres (1). The skin, around puberty, is affected by papules in flexural areas and these alterations are the first clinical signs that are investigated and diagnosed by dermatologists. Skin plaques and/or skin laxity may further develop during disease progression (2). Ophthalmological manifestations (i.e., *peau d'orange*, angioid streaks, and comet lesions) remain clinically silent for at least three decades. Choroidal-neovascularization (CNV) and possibly also dystrophy and atrophy are added over time leading to progressive loss of visual acuity (3, 4). Vascular complications are represented by peripheral artery disease, *claudication intermittent* and in few cases by stroke, transient ischemic attack, and heart attack (5).

PXE patients are typically carriers of two pathogenic variants in the *ABCC6* gene, even though in ~10% of clinically affected patients only one sequence variant can be detected. Moreover, there are a number of pathologic conditions, overlapping the PXE phenotype, where other genes, as γ-glutamyl carboxylase (*GGCX*) or ectonucleotide pyrophosphatase/phosphodiesterase 1 (ENPP1), have a pathogenic role (6–8).

In addition, the heterogeneity of the PXE phenotype in terms of number of organs involved, of disease onset and severity suggested the involvement of modifier genes (9).

Within this context, PXE diagnosis as well as patients' counselling can benefit from the use of next-generation sequencing technologies allowing to facilitate and to broaden the identification of genes that can be involved in the disease.

## Case Report

**Figure 1:**
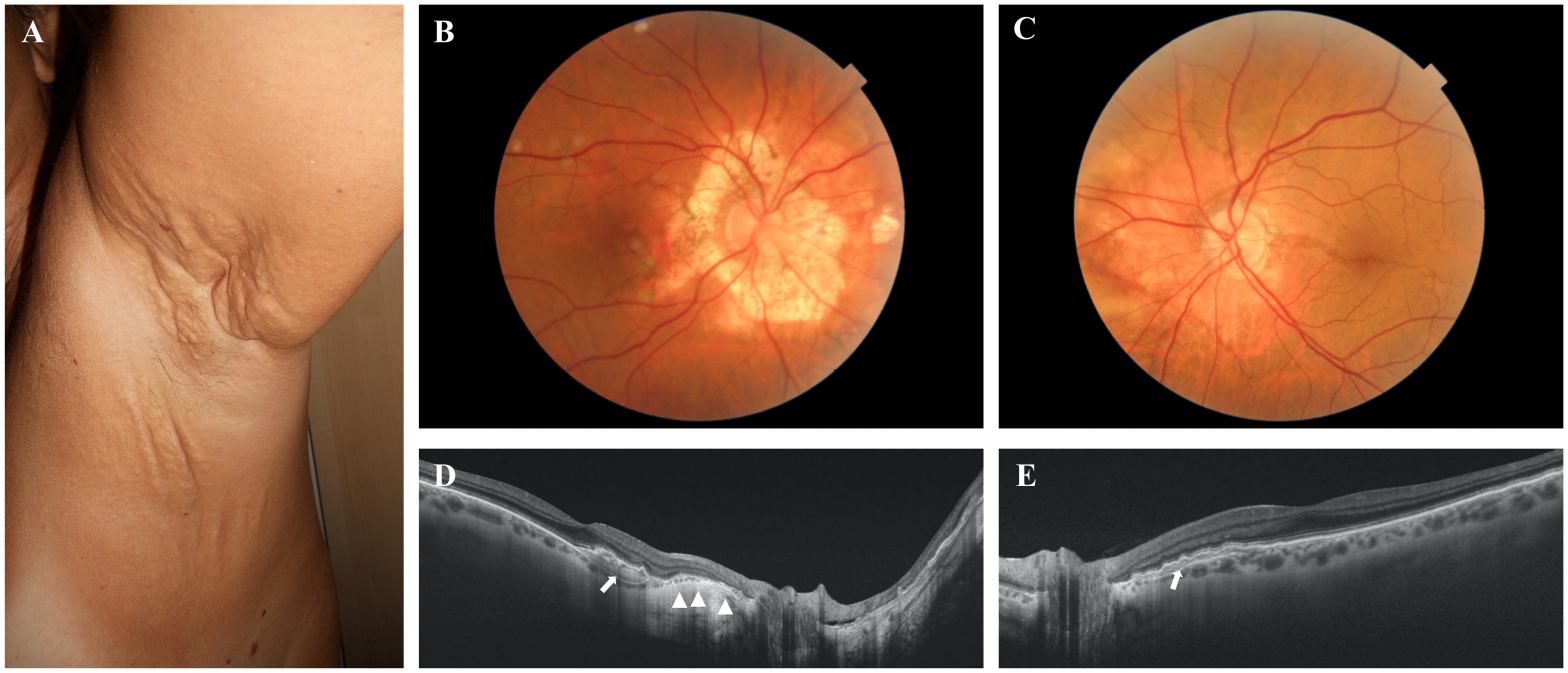
**(A)** Skin plaques and skin laxity in the axillary area. **(B,C)** Colour *fundus* photographs of the posterior pole of the right and left eye, respectively. A large peripapillary atrophic area **(B)** and angioid streak passing through the fovea **(C)** are evident. **(D,E)** Optical coherence tomography examinations of the right and left eye, respectively. The atrophy of the retinal pigment epithelium (RPE) is evident in the right eye (arrowheads). Arrows show the abnormalities of the RPE–Bruch's membrane complex.

A 56-year-old female showed papules on neck and axillae as well as marked skin laxity and redundance (i.e., neck, axillae, periumbelican area, groyne, and inner thighs) (Figure 1A). Ophthalmological examinations revealed angioid streaks and peau d'orange. The right eye exhibited CNV requiring 2 treatments with intravitreal anti-VEGF injections, a large peripapillary atrophy and a small iuxtafoveal scar. Optical coherence tomography examination demonstrated also retinal pigment epithelium (RPE)-Bruch's membrane complex abnormalities nasally to the fovea (Figures 1B–E).

Clinical laboratory tests (i.e., alkaline phosphatase, calcium, phosphorus, magnesium, zinc, iron and blood clotting factors) were within reference ranges.

Unexpectedly, molecular analysis of the ABCC6 gene by Sanger sequencing detected only a monoallelic pathogenic variant (c.4198G>A) (10). Multiplex ligation-dependent probe amplification was negative.

In order to investigate the involvement of other genes, we conducted a whole exome sequencing analysis, performed as already described (11), focusing on 362 genes of which 41 (Supplementary Table 1) have been associated with calcification-related diseases, according to UniProtKB, or with the mineralization process (8, 9), whereas 321 genes (RetNet, https://sph.uth.edu/retnet/) were known as causative of inherited retinal diseases (e.g., macular degeneration, Stargardt disease, retinal atrophy). Using a human genomic variant search engine (VarSome; https://varsome.com) (12), detected rare sequence variants (RSV) were classified as benign, likely benign, uncertain significance, likely pathogenic and pathogenic, according to the guidelines of American College of Medical Genetics and Genomics and the Association for Molecular Pathology (13). RSVs classified as benign or likely benign are not shown. Furthermore, for each detected RSV, an additional search was also performed in PubMed looking for functional studies of altered sequences and for the occurrence of known gene interactions.

Table 1 shows rare sequence variants found in patient.

**Table 1:** List of rare sequence variants found in patient in heterozygous state.

**Gene/Chr–Exon**	**Nucleotide/Amino acid change**	**Freq ExAC**	**Freq GnomAD**	**Freq 1000G**	**dbSNP**	**VarSome**	**Calcification-related genes**	**RetNet gene**	**References**
ABCA4 Chr1–Ex13	c.1928T>G p.Val643Gly	0.001346	0.00172	0.0010	rs61754024	LP		✓	(14)
ABCC6 Chr16–Ex29	c.4198G>A p.Glu1400Lys	0.000009	/	/	rs63751241	P	✓	✓	(10)
AGBL5 Chr2–Ex12	c.2227T>G p.Ser743Ala	0.000058	0.00006	/	rs779635578	VUS		✓	This study
CLUAP1 Chr16–Ex5	c.465T>A p.Tyr155Ter	/	/	/	/	P		✓	This study
GGCX Chr2–Ex2	c.193A>G p.Met122Val	/	/	/	/	LP	✓		This study
KCNV2 Chr9–Ex1	c.349G>A p.Gly117Ser	0.00017	0.000064	0.0002	rs200353727	VUS		✓	This study
SERPINF1 Chr9–Ex3	c.200G>A p.Arg67Gln	0.000025	/	/	rs753681259	VUS	✓		(15)

*Chr, chromosome; Freq, frequency; ExAC, Exome Aggregation Consortium; GnomAD, Genome Aggregation Database; 1000G, 1000 Genomes; /, no data available; dbSNP, single nucleotide polymorphism database; VarSome, https://varsome.com; LP, likely pathogenic; P, pathogenic; VUS, uncertain significance; RetNet, https://sph.uth.edu/retnet/*.

A new RSV was detected on GGCX (c.193A>G) and was classified as likely pathogenic by VarSome.

Patient was also carrier of a RSV in the Serpin Family F Member 1 gene (SERPINF1) (c.200G>A) that was classified of uncertain significance by VarSome. Interestingly, this RSV determines the substitution of Arg in position 67 with Gln. Searching of the literature revealed that, according to mutagenesis analysis studies, Arg67Gln and Arg69Gln have a biological significance inducing an alteration of the nuclear import of the protein (15).

Furthermore, a detected monoallelic *ABCA4* variant (c.1928T>G) was reported as likely pathogenic in VarSome and in the literature (14, 16).

Finally, a non-sense pathogenic RSVs were found in *CLUAP1* (c.465T>A), whereas missense RSV were detected in the *AGBL5* (c.2227T>G) and *KCNV2* (c.349G>A) genes. These last two variants were classified of uncertain significance by VarSome.

## Discussion and Conclusion

PXE is a multisystemic disease, although dermatologists, due to early cutaneous manifestations, are frequently the first physicians to be involved, and therefore even single case reports are instrumental to deepen the knowledge on the challenging complexity of this rare disease.

In particular, the *GGCX* gene encodes a γ-glutamyl carboxylase, an enzyme required for the activation of vitamin K-dependent proteins (e.g., blood coagulation factors and matrix Gla protein-MGP). The carboxylated form of MGP is a key factor in preventing ectopic calcification and reduced γ-glutamyl carboxylase activity implies a decreased carboxylation of MGP, thus favouring pathologic mineralization. Mutations in *GGCX* have been described in patients with *retinitis pigmentosa, cutis laxa*, and *pseudoxanthoma elasticum*-like skin manifestations (17). Moreover, RSVs in *ABCC6* and *GGCX* have been reported in a family with PXE without coagulation disorder (6). Blood coagulation tests performed on our patient did not reveal decreased levels of vitamin K-dependent clotting factors, consistently with the observation that a single RSV on *GGCX* gene is not sufficient to reduce the clotting factor activity (18). Our data support the occurrence of a *GGCX* and *ABCC6* PXE digenic inheritance (6).

Interestingly, we found a RSV in *SERPINF1* gene, which encodes PEDF (pigment epithelium-derived factor), a molecule that, depending on cell type and tissue context, can modulate the expression of osteogenic genes (e.g., *ALP, Runx2, BMP-2*) as well as angiogenesis, being a potent inhibitor of ocular blood vessels' growth. We have already demonstrated the accumulation of PEDF in the calcified skin (19, 20), and alterations in PEDF expression/secretion have been also correlated to the development of CNV and to the pathophysiology of retinal diseases (21). PEDF is both a secreted extracellular and intracellular protein and mutagenesis studies on Arg residues in position 67 and 69 showed that nuclear import of PEDF is compromised and therefore the anti-angiogenic function of PEDF may be modified (15), further supporting the possible involvement of this molecule in PXE.

The *ABCA4* gene encodes an ATP-binding cassette transporter expressed in the RPE and acting as an inward-directed retinoid flipase. RSVs in this gene determine damage to photoreceptors and to the RPE, causing retinal dystrophies (i.e., from mild *fundus flavimaculatus* to cono-rod dystrophy, age-related macular degeneration-2, *retinitis pigmentosa*-like phenotypes, up to retinal atrophy) (14) that have been also described in PXE patients. A recent study reported a patient with two rare variants in the *ABCC6* gene and homozygosity for *ABCA4* RSV suggesting a synergic interaction causing alterations of RPE and/or of photoreceptor function and mineralization of the Bruch's membrane (22). Although the inheritance of these retinal diseases follows an autosomal recessive pattern, some Authors have suggested that patients with only a single rare *ABCA4* variant can represent a subgroup of age-related macular degeneration or of a late-onset Stargardt's disease (23, 24).

Multigene analysis also detected RSVs in *CLUAP1, AGBL5*, and *KCNV2* genes, which are responsible for Leber congenital amaurosis, *retinitis pigmentosa*, and cone dystrophy, respectively (https://sph.uth.edu/retnet/). At present, these genes and/or their products have been never related to PXE, nevertheless we cannot rule out the possibility that, in combination with mutations in other genes, these variants may play a role in patients' ophthalmological manifestations.

In conclusion, rare sequence variants in *GGCX* and *ABCC6* or in *ABCA4* and *ABCC6* have been sporadically reported in single PXE patients (6, 22), whereas no association has been observed between *SERPINF1* and *ABCC6* genes. For the first time, all these four genes are described in a patient clinically diagnosed as PXE, reinforcing the hypothesis that RSVs in these genes, but possibly also in other genes, may contribute to the development and progression of multisystemic clinical manifestations and to the heterogeneity of the PXE phenotype acting in a complementary and/or synergic manner either as causative or as modifier genes.

Physicians may experience difficulties when facing rare diseases with clinical overlapping phenotypes and obstacles may arise for the appropriate recognition of clinical manifestations and for reaching a final diagnosis. Therefore, within this context, PXE patients, as well as their medical doctor, can benefit from the use of multigene analyses which can: (i) facilitate the identification of rare sequence variants involved in the disease, thus avoiding invasive approaches (i.e., skin biopsy required to reveal elastic fibre mineralization when *ABCC6* test does not confirm clinical diagnosis), as well as expensive and time-consuming diagnostic odyssey before reaching a final diagnosis; (ii) deepen the knowledge on the disease in order to better understand the heterogeneity of phenotypic features and of diseases progression and (iii) improve patients' counselling also in a future perspective of personalised medicine approaches.

## Data Availability Statement

The original contributions presented in the study are included in the article/Supplementary Materials, further inquiries can be directed to the corresponding author/s.

## Ethics Statement

The studies involving human participants were reviewed and approved by Ethics Committees (Project ID: 2018/13014). The patient provided her written informed consent to participate in this study.

## Author Contributions

All authors contributed to the analysis of the data, contributed to manuscript revision, read, and approved the submitted version.

## Conflict of Interest

The authors declare that the research was conducted in the absence of any commercial or financial relationships that could be construed as a potential conflict of interest.

## Publisher's Note

All claims expressed in this article are solely those of the authors and do not necessarily represent those of their affiliated organizations, or those of the publisher, the editors and the reviewers. Any product that may be evaluated in this article, or claim that may be made by its manufacturer, is not guaranteed or endorsed by the publisher.

## Supplementary Material

Supplementary Table 1 List of genes responsible for calcification-related diseases or playing a role in the calcification process.
