# Supplementary material for: From Clinical Diagnosis to the Discovery of Multigene Rare Sequence Variants in Pseudoxanthoma elasticum: A Case Report
Source: Front Med (Lausanne). 2021 Aug 26;8:726856. doi: 10.3389/fmed.2021.726856 (PMC8427021; doi:10.3389/fmed.2021.726856)
Supplement: Supplementary Table 1 — List of genes responsible for calcification-related diseases or playing a role in the calcification process. [file Data_Sheet_1.PDF]

Supplementary Material

From clinical diagnosis to the discovery of multigene rare sequence variants in *Pseudoxanthoma elasticum*: a case report.

Francesco Demetrio Lofaro[1], Dario Pasquale Mucciolo[2], Vittoria Murro[2], Laura Pavese[2], Daniela Quaglino[1], Federica Boraldi[1]

[1] Department of Life Science, University of Modena and Reggio Emilia, Modena, Italy

[2] Department of Neuroscience, Psychology, Drug Research and Child Health, University of Florence, Eye Clinic, Florence, Italy

Table S1. List of genes responsible for calcification-related diseases (i.e., systemic mineralization; brain-, chondral-, vascular-, skin-calcification) or playing a role in the calcification process.

	Gene/ Protein	Protein name	Human calcification-related diseases according to UniProtKB or genes related to calcification process
1	ABCC6/ MRP6	Multidrug resistance-associated protein 6	Pseudoxanthoma elasticum (PXE)<br>Arterial calcification of infancy, generalized, 2 (GACI2)
2	AHSG/ FETUA	Alpha-2-HS-glycoprotein	Inhibitor of calcification
3	ALPL/ PPBT	Alkaline phosphatase, tissue-nonspecific isozyme	Hypophosphatasia (HOPS)<br>Hypophosphatasia childhood type (HOPSC)<br>Hypophosphatasia infantile type (HOPSI)
4	ANKH/ ANKH	Progressive ankylosis protein homolog	Chondrocalcinosis 2 (CCAL2)<br>Craniometaphyseal dysplasia, autosomal dominant (CMDD)
5	APOE/ APOE	Apolipoprotein E	Inhibitor of calcification
6	CA2/ CAH2	Carbonic anhydrase 2	Osteopetrosis, autosomal recessive 3 (OPTB3)
7	CASR/ CASR	Extracellular calcium-sensing receptor	Hypocalciuric hypercalcemia, familial 1 (HHC1)<br>Hypocalcemia, autosomal dominant 1 (HYPOC1)
8	CTC1/ CTC1	CST complex subunit CTC1	Cerebroretinal microangiopathy with calcifications and cysts 1 (CRMCC1)
9	DDR2/ DDR2	Discoidin domain-containing receptor 2	Spondyloepimetaphyseal dysplasia, short limb-hand type (SEMD-SL)
10	ENPP1/ ENPP1	Ectonucleotide pyrophosphatase/phosphodiesterase family member 1	Ossification of the posterior longitudinal ligament of the spine (OPLL)<br>Arterial calcification of infancy, generalized, 1 (GACI1)<br>Cole disease (COLED)
11	FAM20A/ FA20A	Pseudokinase FAM20A	Amelogenesis imperfecta 1G (AI1G)
12	FGF23/ FGF23	Fibroblast growth factor 23	Tumoral calcinosis, hyperphosphatemic, familial, 2 (HFTC2)
13	GALNT3/ GALT3	Polypeptide N-acetylgalactosaminyltransferase 3	Tumoral calcinosis, hyperphosphatemic, familial, 1 (HFTC1)
14	GBA/ GLCM	Lysosomal acid glucosylceramidase	Gaucher disease 1 (GD1)<br>Gaucher disease 3C (GD3C)
15	GGCX/ VKGC	Vitamin K-dependent gamma-carboxylase	Combined deficiency of vitamin K-dependent clotting factors 1 (VKCFD1)<br>Pseudoxanthoma elasticum-like disorder with multiple coagulation factor deficiency (PXEL-MCFD)
16	ISG15/ ISG15	Ubiquitin-like protein ISG15	Immunodeficiency 38, with basal ganglia calcification (IMD38)
17	JAM2/ JAM2	Junctional adhesion molecule B	Basal ganglia calcification, idiopathic, 8, autosomal recessive (IBGC8)
18	JAM3/ JAM3	Junctional adhesion molecule C	Hemorrhagic destruction of the brain with subependymal calcification and cataracts (HDBSCC)
19	KL/ KLOT	Klotho	Tumoral calcinosis, hyperphosphatemic, familial, 3 (HFTC3)

20	LBR/<br>LBR	Delta(14)-sterol reductase LBR	Greenberg dysplasia (GRBGD)
21	MGP/<br>MGP	Matrix Gla protein	Keutel syndrome (KTLS)
22	MYORG/<br>MYORG	Myogenesis-regulating glycosidase	Basal ganglia calcification, idiopathic, 7, autosomal recessive (IBGC7)
23	NOTCH1/<br>NOTC1	Neurogenic locus notch homolog protein 1	Aortic valve disease 1 (AOVD1)
24	NRROS/<br>LRC33	Transforming growth factor beta activator LRRC33	Seizures, early-onset, with neurodegeneration and brain calcification (SENEBAC)
25	NT5E/<br>5NTD	5'-nucleotidase	Calcification of joints and arteries (CALJA)
26	OCLN/<br>OCLN	Occludin	Pseudo-TORCH syndrome 1 (PTORCH1)
27	PCDH12/<br>PCD12	Protocadherin-12	Diencephalic-mesencephalic junction dysplasia syndrome 1 (DMJDS1)
28	PDGFB/<br>PDGFB	Platelet-derived growth factor subunit B	Basal ganglia calcification, idiopathic, 5 (IBGC5)
29	PDGFRB/<br>PDGFRB	Platelet-derived growth factor receptor beta	Basal ganglia calcification, idiopathic, 4 (IBGC4)
30	PPA1/<br>IPYR	Inorganic pyrophosphatase 1	Enhancer of calcification
31	SAMD9/<br>SAMD9	Sterile alpha motif domain-containing protein 9	Tumoral calcinosis, normophosphatemic, familial (NFTC)
32	SERPINF1/<br>PEDF	Pigment epithelium-derived factor	Enhancer of osteoblastic gene expression and mineral deposition
33	SLC20A1/<br>S20A1	Sodium-dependent phosphate transporter 1	Enhancer of calcification
34	SLC20A2/<br>S20A2	Sodium-dependent phosphate transporter 2	Basal ganglia calcification, idiopathic, 1 (IBGC1)
35	SPP1/<br>OSTP	Osteopontin	Inhibitor of calcification
36	STN1/<br>STN1	CST complex subunit STN1	Cerebroretinal microangiopathy with calcifications and cysts 2 (CRMCC2)
37	TNFRSF11B/<br>TR11B	Tumor necrosis factor receptor superfamily member 11B	Paget disease of bone 5, juvenile-onset (PDB5)
38	VKORC1/<br>VKOR1	Vitamin K epoxide reductase complex subunit 1	Combined deficiency of vitamin K-dependent clotting factors 2 (VKCFD2)
39	XYLT1/<br>XYLT1	Xylosyltransferase 1	Desbuquois dysplasia 2 (DBQD2)<br>Pseudoxanthoma elasticum (PXE)*
40	XYLT2/<br>XYLT2	Xylosyltransferase 2	Spondyloocular syndrome (SOS)<br>Pseudoxanthoma elasticum (PXE)*
41	XPR1/<br>XPR1	Xenotropic and polytropic retrovirus receptor 1	Basal ganglia calcification, idiopathic, 6 (IBGC6)

* These genes are reported as disease modifier in UniProtKB.
